# Tempe extract reduces cell damage in the liver and kidneys after intensive physical exercise in rats

**DOI:** 10.14202/vetworld.2020.1510-1516

**Published:** 2020-08-07

**Authors:** I. Nyoman Suarsana, Iwan Harjono Utama, I. Made Kardena

**Affiliations:** 1 Laboratory of Biochemistry, Faculty of Veterinary Medicine, Udayana University, Denpasar, Indonesia; 2 Laboratory of Pathology, Faculty of Veterinary Medicine, Udayana University, Denpasar, Bali, Indonesia

**Keywords:** apoptosis, caspase-3, kidney, liver, physical exercise, tempe

## Abstract

**Background and Aim:**

Cells of the liver and kidneys are perpetually exposed to free radicals from endogenous and exogenous sources. High-intensity physical exercise can induce oxidative stress. This study aimed to determine the effects of tempe extract on cell damage in the liver and kidneys of rats after intensive physical exercise.

**Materials and Methods:**

This study used five experimental groups: T0 (non-exercised control rats), T1 (rats made to exercise by swimming), and T2-T4 (rats made to exercise by swimming treated with 25, 50, and 100 mg/kg body weight tempe extract). The biochemical parameters that were analyzed included blood glucose, aspartate aminotransferase (AST), alanine aminotransferase (ALT), alkaline phosphatase (ALP), blood urea nitrogen (BUN), and creatinine levels. The morphology of liver and kidney tissues was histopathologically and immunohistochemically analyzed.

**Results:**

Tempe extract treatment reduced cell damage in the liver and kidney tissues of rats, characterized by decreased expression of caspase-3. In addition, the ALT, AST, ALP, creatinine, and BUN levels of rats were significantly lower in tempe extract-treated rats than in rats after swimming exercise alone.

**Conclusion:**

Tempe extract is capable of reducing cell damage and apoptosis in the liver and kidney cells of rats after intensive physical exercise and maintaining biochemical properties similar to the normal physiological state.

## Introduction

Animal tissues are perpetually exposed to free radicals from both endogenous and exogenous sources, which can induce oxidative stress. One endogenous factor that contributes to oxidative stress through the production of free radicals during metabolism is intensive physical exercise. Exercise causes stress on the internal physical environment. This change can either be advantageous or disadvantageous, depending on the strength of the free radical induction, as intensive physical exercise is also known to cause muscle fatigue, inflammation, and oxidative stress [1]. Oxidative stress is a state of imbalance between reactive oxygen species (ROS) and antioxidants, which can result in molecular and cellular damage. It has long been known that redox signaling contributes to energy metabolism and subsequent production of ROS [2]. Both animals and humans are unavoidably exposed to ROS in daily life. Endogenous free radicals come from normal energy metabolism, physical exercise, and inflammation, while exogenous radicals come from food eaten, environmental stresses, pathogen infections, pollutants, chemical medicines, xenobiotics, insecticide contamination, and radiation [3]. ROS produced from normal physiological processes, such as aerobic respiration and intensive exercise, cause oxidative damage to biomacromolecules, including protein, lipids, and DNA, which can contribute to cell death [4,5]. Oxidative stress induces cell death through necrosis or apoptosis, leading to the surrounding cells, and tissues being damaged. Apoptosis is facilitated by a family of cysteine protease enzymes called caspases. Caspases are usually present in cells in the form of inactive proenzymes and require limited proteolysis for enzymatic activity [6]. Activated caspase-3 cleaves DNA fragmentation factor-45, which induces DNA fragmentation and subsequent apoptosis [7]. Therefore, oxidative stress can play an important role in tissue and organ damage, disease development [8], and the pathology of many diseases, such as metabolic syndrome, diabetes, atherosclerosis, cancer, and osteoporosis [9]. It was reported that aerobic physical exercise is a primary trigger for the formation of free radicals. Increased production of ROS is an indicator of oxidative stress and can cause extensive damage to cells and tissues, leading to cellular dysfunction [10]. Oxidative stress has been considered a pathological mechanism and contributes to liver injury [11], while systemic oxidative stress also causes brain damage and kidney failure [12]. Liver and kidney cell damage leads to the release of several key enzymes and other compounds into the blood, which can be analyzed to determine the degree of damage to both organs.

Dietary interventions and supplements may help protect cells from damage caused by ROS produced due to continuous intensive physical exercise. According to Yu *et al*. [13], the dietary intervention of exogenous antioxidants can decrease the severity of oxidative stress resulting from physical exercise and boost physiological conditions. However, consumers prefer more natural antioxidants in their diets to reduce oxidative damage without side effects, of which tempe is one. Tempe is a traditional Indonesian food produced through the fermentation of soybeans. Tempe contains the bioactive compound isoflavone in the forms of aglicon and glucoside. Isoflavone compounds also include daidzein, genistein, glycitein, and isoflavone factor II (6,7,4’-trihydroxyisoflavone) [14], which demonstrate high antioxidant activity [15].

Intensive physical exercise requires sufficient energy to increase the body’s metabolism. However, the negative consequence of increased metabolism includes the formation of free radicals, which can be dangerous to liver and kidney cells. This study aimed to determine the effects of tempe extract on liver and kidney cell damage in rats after intensive physical exercise.

## Materials and Methods

### Ethical approval

The procedure of this research has been approved by Ethics Committee of Animals using in Research and Education, Faculty of Veterinary Medicine, Udayana University based on certificate number 0705/UN14.2.9/PD/2018.

### Study period and location

This study was conducted from June to August 2018 at Faculty of Veterinary Medicine, Udayana University, Bali, Indonesia.

### Preparation of tempe extract

Tempe was obtained from a local producer (Denpasar City, Indonesia). Tempe (500 g) was sliced and blended and then submerged in ethanol for 24 h. The resulting solution was centrifuged at 1006 g for 15 min. The supernatant was collected, and ethanol was removed using a vacuum evaporator and a container of water (50°C) until the extract became thick. The thick extract was incubated in a desiccator to remove residual ethanol. This extract was taken from the desiccator and placed in an incubator at 37°C for 24 h to dry before it was ground into flour.

### Phytochemical screening

Phytochemical screening of tempe was performed after drying in an oven at 50°C. Qualitative phytochemical determination of active compounds was conducted using assays that detected the presence of alkaloids, flavonoids, saponins, tannins, steroids, and triterpenoids as described by Velavan [16].

### Animal studies

Twenty-five male Sprague Dawley rats (200-225 g) were housed in individual stables. The rats were randomly divided into five treatment groups: T0 (control group), T1 (rats that were physically exercised by swimming), and T2-T4 (rats physically exercised by swimming and also treated with tempe extracts [25, 50, and 100 mg/kg body weight, respectively]). The rats used were allowed to adjust to their new environment for 1 week before beginning the experiment. During the experiment, rats were maintained at 25°C-30°C and 55-65% humidity. The rats were given access to commercial pellets and tap water *ad libitum*. Tempe extract was orally administered by oral gavage. The administration of the extract was carried out for 1 month (30 days).

### Intensive swimming physical exercise

One hour after the rats were given tempe extract, they were individually exercised through the intensive swimming activity in a water container (50 cm diameter and 40 cm deep, 25°C). The rats swam for 1 h a day, from 8:00 am to 9:00 am. After swimming, rats were placed in their individual stables and drank tap water *ad libitum*. This activity was repeated for 1 month (for 30 days).

### Analysis of biochemical parameters

After the final treatment, rats were anesthetized with ketamine-HCl. The blood was taken through from the orbital sinus and placed in a tube containing EDTA to be analyzed for glucose, alanine aminotransferase (ALT), aspartate aminotransferase (AST), alkaline phosphatase (ALP), creatinine, and blood urea nitrogen (BUN) levels. These levels were analyzed using the following commercial diagnostic kits: Fluitest GLU (Cat. No. 4341), Fluitest AST (Cat. no. 1176), Fluitest ALT (Cat. no. 1186), Fluitest AP (Cat. no. 1622), Fluitest Urea (Cat. No. 4705), and Fluitest Crea PAP (Cat. no. 4281). The analysis was conducted using the procedure recommended by the manufacturers, and all biochemical parameters were analyzed through spectrophotometry using an automated photometer (4020-Hitachi, Boehringer Mannheim).

### Histopathological analysis of liver and kidney tissues

Liver and kidney tissues were dissected and then fixed in a 10% normal formalin buffer for at least 24 h. Tissue samples were cut into small pieces before they were processed using standard methods, namely, dehydration, clearing, infiltration, embedding, cutting, and hematoxylin and eosin (H&E) staining, as described by Kiernan[17]. Stained tissue samples were then observed under 400× using a light microscope and photographed (five fields of view).

### Caspase-3 analysis through immunohistochemical staining

Pieces of the tissues within the glass tissue cassette were incubated with 3% H_2_O_2_ in methanol for 15 min. A 10% bovine serum albumin was then dripped onto the tissues for 45 min at 37°C. After the tissues were washed, they were dripped with caspase-3 monoclonal primary antibody (GeneTex™, GTX110543) diluted 1 in 200. One hour later, Histofine Simple Stain MAX PO was dripped for 30 min at room temperature. The result of the antigen-antibody reaction was visualized using diaminobenzidine for 5 min before counterstaining with hematoxylin. Brown color indicated caspase-3-positive cells. Positive cells were randomly counted in five visual fields, as previously described by Simsek *et al*. [18]. Hepatocyte and tubule-positive cells were counted using a light microscope under 400×.

### Statistical analysis

The data obtained were analyzed using analysis of variance followed by the Duncan test with SPSS version 22 software (SPSS Inc., Chicago, USA).

## Results

### Phytochemical screening of tempe extract

The results of the phytochemical screening test show that tempe contained alkaloids, flavonoids, and tannins, whereas it did not contain saponin, steroids, or triterpenoid (Table-1). Flavonoids are phenolic compounds that are widely found in plant tissues, fruits, seeds, and nuts. Phenolic compounds, such as phenolic acids, flavonoids, and isoflavonoids, have been identified in soybeans [19]. Flavonoids have been reported to possess antioxidant properties and are capable of preventing free radical chain reactions [20].

**Table-1:** Screening of tempe phytochemical.

Components	Reactor	Results
Alkaloid	Wagner	+ (Positive)
	Mayer	+ (Positive)
	Dragendorff	+ (Positive)
Flavonoid		+ (Positive)
Tannin		+ (Positive)
Saponin		− (Negative)
Steroid		− (Negative)
Triterpenoid		− (Negative)

### Blood glucose level analysis

**Figure-1:**
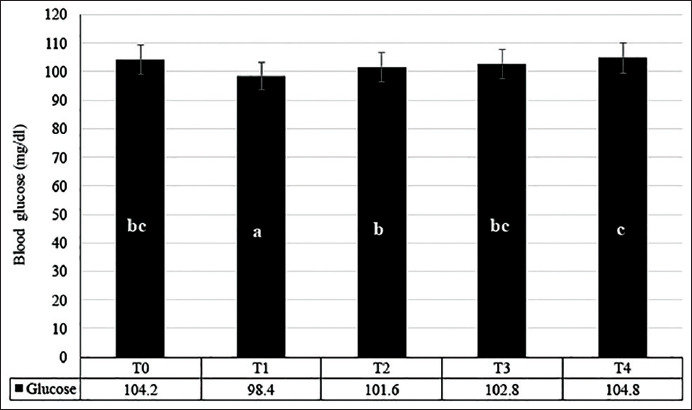
Blood glucose levels of the treated rat with tempe extract and intensive physical exercise for 1 month. T0 = normal; T1 = swimming rat; T2-T4 = swimming and given 25; 50; and 100 mg/kg of tempe extract, respectively.

Blood glucose level is an important parameter of metabolic activity in both active and inactive states. The blood glucose level of rats after swimming exercise (Group T1) was the lowest of all other treatment groups (T0, and T2-T4). The blood glucose level of rats in Group T4 (104.8 mg/dl) was significantly higher (p<0.05) compared to the swimming Group T1 (98.4 mg/dl) (Figure-1).

### Analysis of biochemical parameters

The analysis of biochemical parameters showed that the average value of AST and ALT in the T4 group was 166.8 and 132.4 mg/dl, respectively, which was not significantly different from the control group (T0). However, these activities were significantly lower (p<0.05) compared to rats after the swimming exercise (T1). ALP activity in the T1 group (126.4 mg/dl) was significantly lower (p<0.05) compared to the T0 (144.2 mg/dl), T2 (136.6 mg/dl), T3 (144.2 mg/dl), and T4 (147.4 mg/dl) groups. The average value of creatinine in the control group T0 (0.42 mg/dl) and the rats treated with the highest concentration of tempe extracts along with swimming exercise (T4 group, 0.46 mg/dl) were significantly lower (p<0.05) compared to swimming exercise alone (T1 group, 0.52 mg/dl) or swimming exercise with lower concentrations of tempe extract treatments (T2 group, 0.49 mg/dl, and T3 group, 0.48 mg/dl). The average value of BUN in the control groups T0 (24.5 mg/dl) and T4 (23.5 mg/dl) was also significantly lower (p>0.05) than experimental groups T1 (28.5 mg/dl), T2 (26.4 mg/dl), and T3 (25.7 mg/dl) (Table-2).

**Table-2:** The average value of biochemical parameters of treatment rats of tempe extract and intensive physical exercise for 1 month.

Parameter	T0	T1	T2	T3	T4
Aminotransferase alanine (mg/dl)	124.6±10.3^a^*	149.4±11.2^b^	144.4±10.7^b^	138.6±11.1^a^	132.4±121.1^a^
Aminotransferase aspartate (mg/dl)	157.2±10.1^a^	211.2±12.1^c^	203±11.6^c^	189.4±11.2^b^	166.8±10.8^a^
Creatinine (mg/dl)	0.42±0.02^a^	0.52±0.03^b^	0.46±0.04^a^	0.49±0.03^b^	0.48±0.02^b^
Blood urea nitrogen (mg/dl)	24.5±1.2^a^	28.5±1.6^c^	26.4±1.2^b^	25.7±1.3^b^	23.5±1.4^a^
Phosphatase alkaline (U/L)	144.2±11.1^a^	126.4±11.3^b^	136.6±11.7^a,b^	144.2±12.0^a^	147.4±12.2^a^

* Different letter within same row shows significantly different (p<0.05)

### Histopathological analysis of liver and kidney tissues

Histological analysis of liver and kidney tissues was performed to evaluate the effects of intensive exercise and tempe extract administration. H&E staining was used to observe the morphology of cells of the liver and kidneys.

**Figure-2:**
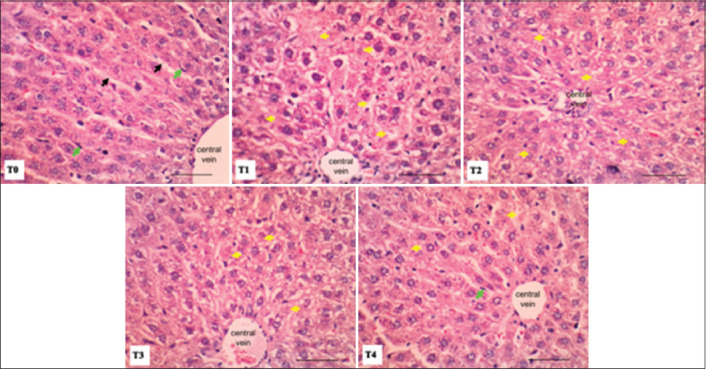
Photomicrograph of rat liver with intensif exercise and tempe extract treatment (Hematoxylin and Eosin staining; bar = 50 µm). Necrosis (yellow arrow), Kupfer cell (green arrow), hepatic sinusoid (black arrow).

Histological changes in the liver are shown in Figure-2. In general, hepatocytes in the sinusoid liver remain clearly visible. However, the intensity of cytoplasmic staining in the liver cells decreased in all treatment groups compared to the control group (T0). Histological analysis of the liver in the exercise group T1 indicated that most of the liver cells exhibited features of necrosis. However, liver cells from rats in treatment groups T2-T3 were observed to have more moderate features of necrosis, while only mild necrosis was observed in the liver cells of the T4 group.

**Figure-3:**
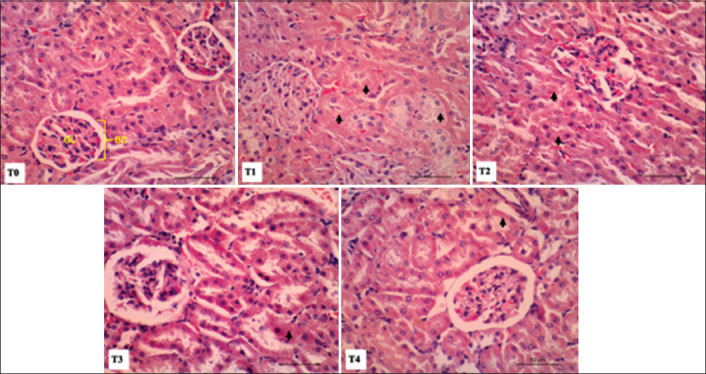
Photomicrograph of rat kidney with intensif exercise and tempe extract treatment (Hematoxylin and Eosin staining; bar = 50 µm). Multifocal necrosis (black arrow), GL: Glomerulus, and BC: Bowman’s capsule.

Histological staining of renal tissue is shown in Figure-3. Photomicrographs of renal tissue in the normal group (T0) showed obvious glomeruli with Bowman’s capsules. However, in the T1 treatment group, degeneration, and renal tubular cell necrosis were observed. Meanwhile, in the T2-T4 treatment groups, necrotic features were observed in the renal cells with a lower necrosis score and significantly different (P<0.05) compared to the T1 treatment group.

### Immunohistochemical staining of caspase-3 in liver and kidney cells

**Figure-4:**
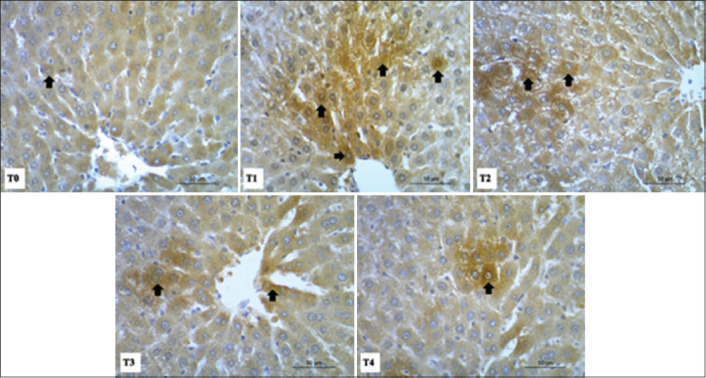
Immunohistochemical staining of Caspase 3 in paraffin section of liver tissues using primary antibody monoclonal anti-caspase 3. Positive Caspase expression is characterized by brown color staining of the cytoplasm (arrows). Highly positive (+++) for caspase-3 as in T1, moderate positivity (++) as in T2-T3, and lower positivity (+) as in T4.

**Figure-5:**
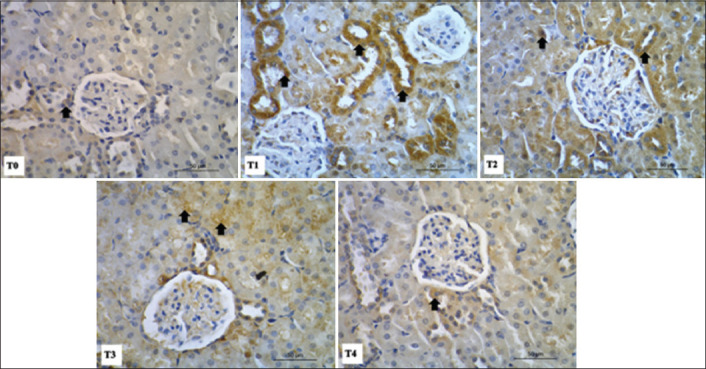
Immunohistochemical staining of caspase-3 in paraffin sections of kidney tissues using monoclonal anti-caspase 3 antibodies. Positive caspase cells are showed by brown color (arrows) which prominently noticed in necrotic tubules tissue. Highly positive (+++) for caspase 3 detected as in T1, moderate positivity (++) as in T2-T3, and lower positivity (+) as in T4.

Caspase-3 expression in cells is an indicator of cell damage and apoptotic death. Primary monoclonal anti-caspase-3 antibodies were used in immunohistochemical staining in liver and kidney tissues. Our results indicated that excessive physical exercise in rats (T1) led to an increase in caspase-3 expression, both in liver (Figure-4) and kidney (Figure-5) tissues.

Apoptotic cells in the liver tissue were observed (Figure-4, brown staining). These cells were distributed in the periphery of necrotic hepatocytes. Caspase-3 expression was marked by high-intensity staining (+++) in T1, moderate intensity (++) in T2-T3, and lower intensity (+) in T4. Incidence of apoptosis in liver tissue of rats after intensive physical activity (T1) was widely distributed in the tissue, as characterized by the expression of caspase-3 spreading in the cytoplasm and in the nucleus of cells. In rats treated with tempe extract (T2-T4), caspase-3 expression was reduced in a manner that correlates with increased tempe extract concentration (Figure-4).

Apoptotic cells also appeared in the renal tubules of kidney tissue. A strong positive caspase-3 signal (+++) was observed in the T1 group, while moderate (++) and lower (+) intensities were observed in the T2-T3 and T4 treatment groups, respectively. In kidney tissue, expression of caspase-3 was highest in the treatment group with intensive physical activity alone (T1), whereas tempe extract led to a dose-dependent decrease in caspase-3 expression following physical exercise (Figure-5).

Liver tissue in the T1 group had the highest number of caspase-3-positive cells, significantly higher (p<0.05) than that in the T0 or T2-T4 groups. The quantity of stained cells of each treatment group as counted from immunohistochemical staining is shown in Table-3. Similar changes were also observed in the kidney tissues.

**Table-3:** The number of expressing caspase-3 in rat liver and kidney tissue.

Treatments	Number of liver cells expressing caspase-3	Number of kidney cells expressing caspase-3
T0	5.12±0.89^a^*	4.16±0.77^a^
T1	27.84±1.89^b^	33.48±1.73^b^
T2	20.44±1.72^c^	28.08±1.75^c^
T3	13.72±1.34^d^	20.68±1.52^d^
T4	8.32±0.95^e^	10.44±1.01^e^

* Different letter within same column shows significantly different (p<0.05)

## Discussion

The primary source of energy used during physical activity is glucose. Glucose is released into the bloodstream to be carried to the muscle and brain cells where it is taken up and used in cellular respiration. Therefore, the blood glucose level is an important indicator of how the body performs during physical exercise [21].

In normal conditions, the physiological and biochemical parameters of blood do not vary wildly. However, these biochemical parameters can fluctuate; for example, they can be increased or decreased when the cell becomes stressed, disturbed, or damaged, and causing dysfunction. Intake of certain compounds can help prevent cellular dysfunction resulting from wounds or excessive physical exercise.

Intensive physical exercise can cause imbalance between oxidation and antioxidation within cells [22], resulting in free radical accumulation and subsequent lipid, protein, and DNA damage. In this study, the biochemical parameters measured on intense physical activity (swimming) in rats were higher than those of the control group (Table-1). This indicated that the cellular function was disturbed, as enzymes within the cells were released into the blood vessels. This cellular disturbance occurred within different organs, including the liver, kidney, and muscle cells. The high activities of AST, ALT, and ALP observed in group T1 are indicative of disturbance of liver and kidney cells. The high creatinine levels observed in this group also indicated the excessive metabolic activity of muscle cells, which led to the production of excessive creatinine. The increased BUN levels could also indicate that kidney function had been disturbed.

High concentrations of tempe extract fed to rats decreased biochemical parameters to levels resembling those in control animals, demonstrating that the bioactive compounds in tempe extract could maintain the performance of the cell despite the effects of intensive physical exercise, such as swimming. Chiang *et al*. [23] claimed that these bioactive compounds could function as adaptogens or compounds that can normalize cell tolerance to stress conditions. Apart from nutritious value, tempe also contains isoflavone compounds. Isoflavone in soybeans includes daidzein, genistein, and glycitein [24]. Tempe also contains 6,7,4’-trihydroxyisoflavone. Isoflavone shows a high antioxidant activity [15], which functions to neutralize free radicals to prevent and reduce damage to liver and kidney cells.

Free radicals produced during intensive physical exercise caused liver and kidney cell necrosis and degeneration. During physical exercise, ROS were excessively produced, leading to oxidative stress. Excessive accumulation of ROS leads to the modification of biomolecules, such as lipids, proteins, and DNA; as a result, the cell function progressively worsens, finally damaging tissues and organs [25]. ROS induces cell death through necrosis or apoptosis [11].

Tempe extracts fed to rats before intensive physical exercise could reduce oxidative stress through the active mechanism of isoflavone compounds in antioxidative free radical scavenging. Tempe extract could limit the chain reactions of free radicals by reducing their initial formation; in this way, the cells could prevent damage, as can be seen from the biochemical parameters in groups T4 and T0.

Apoptosis, or programmed cell death, is important for the normal functioning and survival of most multicellular organisms [26]. However, apoptosis can be accelerated as a result of free radical exposure. Recent studies have demonstrated that ROS and oxidative stress resulting from intensive physical activities play an important role in apoptosis. ROS, together with their effect on cellular redox status, can participate in signal transduction during apoptosis. Furthermore, apoptosis occurs on formation of the multimeric complex of cytochrome C, APAF-1, and caspase 9, which activates downstream caspases to induce apoptotic cell death.

Tempe extract has been shown to prevent cell death due to apoptosis. The mechanism of preventing cell death caused by ROS may be through the ROS scavenging activity of isoflavones and other antioxidants. According to Megowan *et al*. [27], this argument is further supported by the use of other various antioxidants, such as N-acetylcysteine, to block apoptosis in a similar manner as caspase inhibitors.

The results of tempe extract administration in the T4 treatment group demonstrated that tempe extract can prevent liver and kidney damage through serum biochemical parameters, histological analysis, and immunohistochemical analysis of caspase-3. Therefore, in the future, tempe extract may be developed as a powerful natural source of antioxidants for the prevention of cellular oxidative damage in aging or degenerative disease pathology.

## Conclusion

Tempe extract can reduce cell damage and apoptosis in rat liver and kidneys induced by intensive physical exercise. Tempe extract can help maintain the biochemical parameters of AST, ALT, ALP, creatinine, and BUN in the normal physiological range.

## Authors’ Contributions

INS designed and organized the research project, data analysis, and scientific article writing. IHU and IMK carried out the laboratory tests and gathered the research results, analyzed the data, and edited the manuscript. All authors drafted and approved the final manuscript.

## Competing Interests

The authors declare that they have no competing interests.

## Publisher’s Note

Veterinary World remains neutral with regard to jurisdictional claims in published institutional affiliation.
